# Supplementary material for: Common mental health problems and associated factors among recovered COVID-19 patients in rural area: A community-based survey in Bangladesh
Source: PLoS One. 2024 Apr 1;19(4):e0294495. doi: 10.1371/journal.pone.0294495 (PMC10984393; doi:10.1371/journal.pone.0294495)
Supplement: S1 Table — (DOCX) [file pone.0294495.s001.docx]

**Supporting Information**

**S1 Table. DASS 21 subscales for scores for depression, anxiety, and stress**

| **Subscale** | **Depression** | **Anxiety** | **Stress** |
| --- | --- | --- | --- |
| Normal | 0-9 | 0-7 | 0-14 |
| Mild | 10-13 | 8-9 | 15-18 |
| Moderate | 14-20 | 10-14 | 19-25 |
| Severe | 21-27 | 15-19 | 26-33 |
| Extremely Severe | 28+ | 20+ | 34+ |
