# Supplementary material for: Common mental health problems and associated factors among recovered COVID-19 patients in rural area: A community-based survey in Bangladesh
Source: PLoS One. 2024 Apr 1;19(4):e0294495. doi: 10.1371/journal.pone.0294495 (PMC10984393; doi:10.1371/journal.pone.0294495)
Supplement: Attachment — Submitted filename: Response to reviwers.docx [file pone.0294495.s002.docx]

Q1. Metal health is broad concept and the instrument they used to address the concept mainly is for anxiety, depression, and stress. Having this thought in mind I will frame the title as Common mental disorders rather than mental health.

Answer: Thanks for the suggestions, we completely agree with your point. We have changed the title as “Common mental health problems and associated factors among recovered COVID-19 patients in rural area: A community-based survey in Bangladesh”
Introduction
Q2. Page 10 line 70-74 … you mentioned a previous study showing higher prevalence of MH among people recovered from COVID in Bangladesh. If there is a previous study showing the problem, then what is new in this study?

Answer: Thanks for the query. The other study has been completed through online survey by a convenience sample recruited via social media whereas as, in this current study data has been collected data by face to face interview from a rural community.
Q3. All in all, I am not well convinced in the introduction. The main part of this study is MH, however, most of the paragraphs in the study are about pandemics before COVID (which I don’t like). I will recommend reframing this section focusing on only COVID and Mental health and showing what new things that this study is going to address.

Answer: Edited. I have rewritten the introduction part according to your valuable suggestion.

Methods
Q4. Methodologically I haven’t seen any mechanism that showed the effect of COVID on MH of the participants. I will have a control group without COVID so that I will have control over the effect of COVID alone. The prevalence reported here may not attributed to COVID only.

Answer: Edited accordingly.

Q5. On the statistical analysis section the specific analysis technique used in multivariable level analyse was not mention (they only said in the final model we do …..). They also used in appropriate terminologies that does not fit to the objective of the study like multivariate analysis. For me, multivariate analyses are those analysis which involve interplay of multiple variables such as Factor analysis. I recommend revising this section based on the points I raised here. Furthermore, they said they have a cut-off value of <0.2 to decide variable for multivariable analyse. It looks that the researchers are looking for association blindly (without having a prior hypothesis). For me, variables to be included into the final model should be theory driven rather than statistic driven.

Answer: Edited accordingly
Result
Q6. Table 1: it will be good if the level of significance in terms of p-values will be added as one column (as this stand it gave little information.

Answer: Edited accordingly

Q7. Figure 1: the title should be below the figure and also, I will use no depression rather than normal
the format of Table is not attractive, either reduce information or the font size of the table so that it will be attractive for readers.

Answer: Edited accordingly
Discussion
Q8. The prevalence of mental health reported is about 40% which is considered very high, this might be due to COVID but as I already said in the methodology section the effect of the COVID is not well controlled. Therefore, it is difficult to infer it to the pandemic (I don’t agree with the discussion). Finings such as higher prevalence among Female, and young participants and protective effect of good support system (family support, marital status, educational, and economic status), support the above point that in general population CMD is more prevalent in female and youngsters and lower odds in those who had good social support (still not convinced to attribute it to COVID).

Answer: Edited Accordingly.
Conclusion
Q9. It is not advised to use terms like risk in cross-sectional study. Rather use terms like increased odds and like.

Ans: Edited accordingly

Q10. Reviewer #2: First two sentences in the abstract are broad/general with no clear link to mental health. Further in the introduction section, line 49-51 – “As the evidence on its impacts continues to expand, it is becoming increasingly evident that the pandemic has had profound influence on the psychological health of the entire societies due to the changes in daily routines [3]. These mental health issues have been given greater attention in high-income countries” - The first statement is too crude – how the pandemic would have ‘psychological health’ on the entire society; what changes of daily routines should be clarified. The inconsistencies on ‘psychological health’ and ‘mental health issues’ is also worrying. What is the argument here?

Answer: Edited accordingly

Q11. Line 53-56 “…the severity of mental health problems in these parts of the world would have even greater detrimental consequences, given that in most LMICs the resources designated for mental health are limited (and can be below 1% of the health budget)” – Although this may be logically true, I am wondering if there are more recent evidences than the 2017 publications referred here?

Answer: Edited accordingly
Q12. Reducing the stigma of mental health disorders with a focus on low- and middle-income countries the objective of the study is not clear – what is the intension of the manuscript? What is intended other than bridging the ‘gap’ which in itself is not clearer in terms of what gap it refers to.

Answer: Edited accordingly

Q13. Line 79-81 in the intro states – “Thus, there is a paucity of data that reveal the prevalence of depression, anxiety, and stress among Bangladeshi patients that have recovered from COVID-19” – Does this make sense when the title is about the status of mental health status of … So, it is important to spell out what objective is intended to be addressed.

Answer: Thanks for the suggestions, we completely agree with your point. We have changed the title,
Q14. Method – Selecting participants - how long after recovery? This may have good implication on ‘metal health issues’ Line 89-92 – “After excluding children and patients with active infection (within 14 days after diagnosis), 320 recovered COVID-19 patients remained, and we contacted each individual over the phone to invite them to take part in the study. As 243 of these individuals agreed to an interview, the non-response rate was 24%.”

Answer: Edited accordingly
Q15. Firstly, what if the patient did not recover after 14 days for there are those with COVID who may take extra days to get cleared? Secondly, how could the high nonresponse rate could affect your result – not indicated in the limitation section?

Answer: Edited accordingly
Q16. I am wondering if the Bengali version of the Depression, Anxiety, and Stress Scale (DASS-21) was checked if it indeed measures what it is supposed to measure. Am wondering if cronbach alpha was applied to that effect or how else was tool validated for this particular study? Mention was made on line 107 “The validated Bengali version was thus used in this study” while it is not clear on who validated it and by whom – no reference was provided.

Answer: Edited accordingly

Q17.Line 148-149 – “To examine the associations of sociodemographic factors with the participants’ mental health state, Chi-squared, Mann–Whitney, or t-tests were performed, as appropriate” – Which test was applied under what circumstance need to be clearly pointed out. “When appropriate’ may affect the assessment of relevance of test.

Answer: Edited Accordingly
Q18.The statistical methods applied in this study especially the logistic regression as indicated on lines 150-153 do not seem to fit at least to the title which is about the status of mental health instead of associated factors nor do we have this clearer in the objective section. There is no reason/justification for why prediction was considered.

Answer: Edited Accordingly
Q19. Result section did not clarify if depressive symptoms, stress symptoms and anxiety are considered as long term ‘mental health issues. While the standard tool may have defined as such, this has to be well articulated here and perhaps later in the introduction.
What is the association between some (selected??) co-morbidities, BCG inoculation and blood type was not clear and association of these to the objective of the study? This was not clearer?

Answer: Edited accordingly.

Q20.Discussion is fairly ok although with clearer objectives and subsequent restructuring of the result section, discussion section may improve.

Answer: Thanks for your kind concern. Edited accordingly.

Q21.The conclusion section gives an impression that in Bangladesh, psychological health is not given sufficient attention, improvement of mental health services need to improve… that do not have backing from the result. This needs to be re-written.

Answer: Edited accordingly.
