# Supplementary material for: Common mental health problems and associated factors among recovered COVID-19 patients in rural area: A community-based survey in Bangladesh
Source: PLoS One. 2024 Apr 1;19(4):e0294495. doi: 10.1371/journal.pone.0294495 (PMC10984393; doi:10.1371/journal.pone.0294495)
Supplement: Attachment — Submitted filename: Response to Reviewers_2.docx [file pone.0294495.s003.docx]

**Response to Reviewers**

Reviewer #2: a) findings show that 24%, 21% and 22% have exhibited depressive symptom, stress and anxiety respectively. Given there is no evidence on when after recovery was the interview made, this finding could probably suffer much way beyond the recall bias. What if they do not even remember of how they felt about those then?

Answer: The interviews were conducted with individuals who had recovered from COVID-19 between March and September 2020. Specifically, our focus was on patients whose recovery occurred approximately 14 days after contracting the virus. Participants were instructed to reflect on their experiences during the preceding week. Our study has emphasized the participants' feelings and experiences during this specific period. So, duration from recovery to time of interview has no connection.

b) 24% non-response is quite a large number despite consideration of convivence sampling. This warrants proper explanation and proper documentation in the limitation section.

Answer: Edited accordingly in the limitation section. A significant limitation of this study is the substantial non-response rate of 24%, despite the utilization of convenience sampling, which could be attributed to the prevailing stigma associated with mental health issues among people in Bangladesh. This cultural stigma might have made individuals apprehensive about disclosing their mental health-related information. Additionally, the period during which the study was conducted coincided with a relatively high death rate, possibly stemming from various health-related issues and external circumstances. Furthermore, the imposition of lockdown measures, possibly due to the prevailing circumstances, could have further hindered individuals' willingness and ability to participate in our study.

Reviewer #3: This is a good research; however, based on this study, the author can include several valuable recommendations to ensure that they can make the most out of this research. Such suggestions include:
Remote Mental Health Services: Given the challenges posed by the pandemic, remote mental health services like teletherapy and virtual support groups could play a significant role in reaching a broader population of recovered patients, particularly those in remote or underserved areas.
Collaboration with Global Health Organizations: Partnering with international health organizations and experts could offer valuable insights and resources for enhancing mental health services in Bangladesh. Sharing best practices and lessons learned from other countries can contribute to more effective interventions.
Policy Reforms and Funding Allocation: The urgency of addressing the mental health crisis should prompt policymakers to reevaluate resource allocation and policy reforms to prioritize mental health services. Adequate funding and policy support are essential for establishing a sustainable and effective mental health infrastructure.

Answer: Thank you for your valuable suggestion. Recommendations are added according to your suggestions.
