# Supplementary material for: Common mental health problems and associated factors among recovered COVID-19 patients in rural area: A community-based survey in Bangladesh
Source: PLoS One. 2024 Apr 1;19(4):e0294495. doi: 10.1371/journal.pone.0294495 (PMC10984393; doi:10.1371/journal.pone.0294495)
Supplement: Attachment — Submitted filename: Response to Reviewers_3.docx [file pone.0294495.s004.docx]

To

The Editor

PLOS ONE,

Subject: Response to Reviewers for Manuscript ID PONE-D-22-13763R2, titled Common mental health problems and associated factors among recovered COVID-19 patients in rural area: A community-based survey in Bangladesh"

Dear Sir,

I would like to express my sincere gratitude to you and the reviewers for providing invaluable feedback on our manuscript. We greatly appreciate the time and effort invested in evaluating our work. We have carefully addressed each of the comments and suggestions made by the reviewers, and I am pleased to provide a detailed response below.

We are pleased to note that Reviewer #2 found that all of their previous comments were adequately addressed. We would like to express our gratitude for their careful consideration of our revisions.

Reviewer #2 has confirmed that the manuscript is technically sound, and the data robustly support our conclusions. We are delighted to receive this validation, and we believe this strengthens the overall quality of our research.

The reviewer also commended the appropriateness and rigor of our statistical analysis. We thank them for their careful scrutiny of this aspect of our work.

Reviewer #2 has noted that the manuscript is presented in an intelligible fashion and written in standard English. We are grateful for their attention to detail in this regard, and we have made sure to correct any typographical or grammatical errors during the revision process.

We would like to extend our sincere appreciation to Reviewer #3 for their kind words and thorough review of our research. We are pleased to know that they found our revisions to be comprehensive and that they acknowledge the dedication and expertise demonstrated by our team.

We are honored by Reviewer #3's commendation of the meticulous work and the high level of dedication and expertise displayed in our research. Their positive assessment reaffirms our commitment to producing high-quality work.

Reviewer #3 has emphasized the significance of addressing recommendations rigorously, and we are grateful for their recognition of our efforts in this regard. We are pleased that our research sets a positive example for others in the field.

In summary, we are grateful to both reviewers for their positive feedback and for finding that all of their previous comments and queries were thoroughly addressed. Their assessments have been instrumental in refining our manuscript and enhancing its overall quality.

We would like to express our consent to publish the peer review history of our article, and we have indicated our preference for public attribution.

Once again, we extend our gratitude to you and the reviewers for their time and expertise. We look forward to hearing from you regarding the next steps in the publication process.

Best regards,

**M Atiqul Haque (Ph.D.)**

Professor

Department of Public Health and Informatics,

Bangabandhu Sheikh Mujib Medical University, Shahbag, Dhaka-1000

Email: [atiqulm26@bsmmu.edu.bd](mailto:atiqulm26@bsmmu.edu.bd)
